# Identification and Field Evaluation of Sex Pheromone Components and Its Antagonist Produced by a Major Tea Pest, *Archips strojny* (Lepidoptera: Tortricidae)

**DOI:** 10.3390/insects13111056

**Published:** 2022-11-15

**Authors:** Nanxia Fu, Fida Hussain Magsi, Yingjie Zhao, Xiaoming Cai, Zhaoqun Li, Lei Bian, Chunli Xiu, Zongmao Chen, Zongxiu Luo

**Affiliations:** 1 Tea Research Institute, Chinese Academy of Agricultural Sciences, Hangzhou 310008, China; 2 Key Laboratory of Biology, Genetics and Breeding of Special Economic Animals and Plants, Ministry of Agriculture and Rural Affairs, Hangzhou 310008, China

**Keywords:** female sex pheromone, *Archips strojny*, (*Z*)-11-tetradecenyl acetate, male attraction, tea pest

## Abstract

**Simple Summary:**

The tea tortrix *Archips strojny* is a major pest of spring tea in China. Using sex pheromone-based mass trapping or mating disruption techniques to control this pest can minimize excessive pesticide usage, thereby maintaining pesticide residues within the safety standard for spring tea products. However, the sex pheromone components of this species remain elusive. In this study, we identified these components: laboratory assays showed that two bioactive compounds in female sex pheromone gland extracts, namely, (*Z*)-11-tetradecenyl acetate (Z11-14:Ac) and (*Z*)-11-tetradecenyl alcohol (Z11-14:OH), could elicit male moth antennal responses. A field trapping assay demonstrated that Z11-14:Ac was the attractant in the sex pheromone, while Z11-14:OH seemed to be an antagonist. Overall, our results indicated that lures baited with 1 mg of Z11-14:Ac could be used as a monitoring or mass trapping tool for *A. strojny* management in Chinese tea plantations. Furthermore, Z11-14:Ac was found to be a common attractant in the sex pheromones of nine leafroller species in the genus *Archips*. Thus, Z11-14:Ac may be used to develop a mating disruption technique targeting multiple leafroller species.

**Abstract:**

Pesticide application is the only known control method for the tea tortrix *Archips strojny* (Lepidoptera: Tortricidae), which is a major pest of spring tea in China. To develop sex pheromone-based, environmentally safe control strategies, here we identified the sex pheromone components of this species. The male moths’ antennae responded electrophysiologically to two compounds in female pheromone gland extracts. Gas chromatography–mass spectrometry analysis indicated that the two bioactive compounds were (*Z*)-11-tetradecenyl acetate (Z11-14:Ac) and (*Z*)-11-tetradecenyl alcohol (Z11-14:OH). Field trapping assays showed that lures baited with only the major component Z11-14:Ac were the most attractive to male moths, and the attractiveness decreased significantly when the lure was impregnated with increased relative ratios of the minor component Z11-14:OH. Our study demonstrated that Z11-14:Ac was the major attractant in the *A. strojny* sex pheromone, and the minor component Z11-14:OH seemed to serve as an antagonist. The results indicate that lures baited with 1 mg of Z11-14:Ac could be used as a monitoring or mass trapping tool for *A. strojny* management in Chinese tea plantations. Furthermore, Z11-14:Ac was identified as a common sex pheromone attractant of nine *Archips* species; these results lay the foundation for developing mating disruption techniques that target multiple leafroller pests.

## 1. Introduction

Tea is one of the most popular non-alcoholic beverages in the world, and it has been consumed for thousands of years [1,2]. The tea plant *Camellia sinensis* Kuntze, which originated in southwest China, is now cultivated as an important commercial crop in more than 40 countries, predominantly in Asia [3]. The tea tortrix *Archips strojny* Razowski is one of the most destructive pest species in Chinese tea plantations [4,5,6,7,8,9]. Tea tortrix larvae roll up the tea leaves into a tube shape to hide and feed inside; this concealment makes early detection and control of these pests in the larval stage difficult. *Archips strojny* is one of a number of tea tortrix species in the family Tortricidae, which has recently been found to spread widely in tea plantations in China [10]. Each year, the first generation of *A. strojny* larvae occurs in early April and severely damages the tender tea leaves, causing substantial yield losses [10]. In China, spring tea is preferred by consumers because of its umami taste and pleasurable aroma [11,12]. As the harvest season for spring tea is known to coincide with the peak of the *A. strojny* infestation period, the use of pesticides leaves harmful chemical residues in spring tea products, resulting in economic losses for tea farmers [10]. It is thus essential to design environmentally friendly strategies to control this tea pest. Semiochemicals are highly efficient alternative tools for monitoring and managing pest populations and have been used to manage other destructive moth species on a large scale [13].

Sex pheromones are species-specific volatile chemicals, usually produced and released by female insects to attract conspecific males [14]. Because of their species-specificity and non-toxicity to mammals and beneficial insects, sex pheromones have been widely used as an effective and environmentally friendly method for pest population monitoring, mating disruption, and mass trapping [15,16,17]. The sex pheromones of approximately 176 species belonging to the Tortricidae family have been identified, and the majority of them produce Type I sex pheromones, namely, C10–C18 unsaturated straight chains with a terminal functional group, such as a hydroxyl, acetoxyl, or formyl group [18,19]. For instance, the smaller tea tortricid *Adoxophyes honmai* Yasuda emitted (Z)-9-tetradecenyl acetate (Z9-14:Ac) and (Z)-11-tetradecenyl acetate (Z11-14:Ac) as a sex pheromone [20], and female oriental tea tortrix *Homona magnanima* Diakonoff secreted Z11-14:Ac, (Z)-9-dodecenyl acetate (Z9-12:Ac), and 11-dodecenyl acetate (11-12:Ac) to attract their male counterparts [21]. In Japanese tea plantations, a commercial mating disrupter composed of the common component Z11-14:Ac was applied to control both *A. honmai* and *H. magnanima* [7,22]. This successful mating disruption strategy has become a classic case of using sex pheromones for pest management.

The application of semiochemicals not only provides an alternative management tool but also minimizes the risk of pesticide residues [13,23]. The use of semiochemicals such as sex pheromones will be highly beneficial, especially in the case of tea products, which are considered to be healthy beverages by consumers around the world [24,25]. The main research objective of our study was the identification of chemical communication compounds of *A. strojny* for the development of a sustainable, environmentally safe prevention management strategy. However, the sex pheromone of *A. strojny* has not yet been investigated; therefore, to develop a pheromone-based management strategy, we aimed to identify the components and structures of the sex pheromone of this species. We extracted the crude pheromone of *A. strojny* from adult females, identified the pheromone components and their structures with a combination of gas chromatography and an electroantennographic detector (GC–EAD), and GC combined with mass spectrometry (GC–MS). Finally, the attractiveness of the identified pheromone compounds of *A. strojny* was evaluated using field trapping assays.

## 2. Materials and Methods

### 2.1. Insects

**Figure 1:**
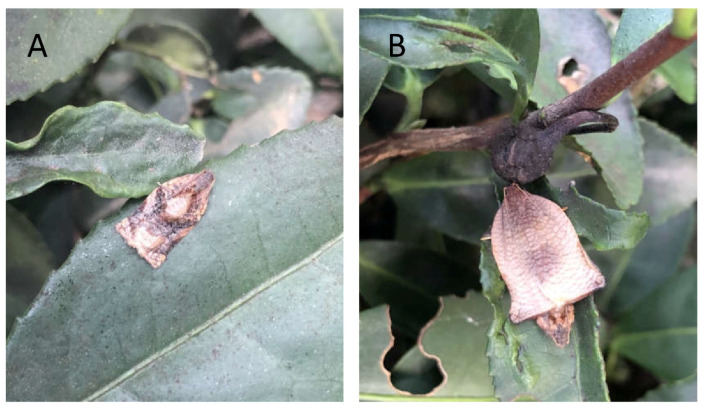
Tea tortrix *Archips strojny* on tea plants. (**A**) Male moth, (**B**) female moth.

Tea tortrix moths *A. strojny* were captured from a tea plantation in Xihu District in Hangzhou City, Zhejiang Province, China (30.17° N, 120.03° E) on 16 March 2020 (Figure 1). Male and female moths were separated into different cages (60 cm × 60 cm × 60 cm) and were reared in a 16:8 h light–dark cycle at 25 ± 1 °C and 60–70% relative humidity. To obtain offspring for the sustainability study, 30 pairs of male and female *A. strojny* moths were reared together in the same mesh cage, supplemented with 5% honey water.

### 2.2. Pheromone Extraction

Active and healthy field-collected female moths were selected for sex pheromone extraction. Pheromone extraction was conducted 4 h after the onset of scotophase, corresponding to the peak calling time of female *A. strojny*. To expose the pheromone gland, the abdomen of a female moth was gently squeezed. The extruded eighth and ninth abdominal segments were excised using a pair of surgical scissors, and 30 glands were immersed in 150 μL of n-hexane in a glass vial. After 20 min, extraction was performed at ambient room temperature. Briefly, the supernatant was first concentrated under nitrogen gas flow and then transferred to a brown vial (Agilent Technologies, Santa Clara, CA, USA). In total, 120 female pheromone glands were pooled as one pheromone sample. Finally, all samples were stored at −20 °C for subsequent analysis.

### 2.3. Gas Chromatography–Electroantennographic Detection (GC–EAD)

To identify olfactory stimulants in the female pheromone samples, an Agilent 7890 gas chromatograph equipped with an HP-5 column (30 m length × 250 µm inner diameter × 0.25 µm film thickness; J&W Scientific, Agilent Technologies, Santa Clara, CA, USA) coupled with electroantennographic detection (EAD) (Syntech, Buchenbach, Germany) was used to record male antennal responses. Tips of both the distal and basal segments of antennae from healthy male *A. strojny* moths were clipped. Then, the antennae were connected with two glass electrodes filled with 0.9% NaCl solution. In total, five male antennae were tested. For each test, 1 μL (equal to extracts from eight female pheromone glands) of concentrated crude female pheromone gland extract was used for analysis. Helium was used as the carrier gas at a flow rate of 1.2 mL/min. The injector temperature was 280 °C, and the column oven was initially held at 60 °C; then the temperature was raised by 10 °C /min to 250 °C and held for 2 min. The injected sample was introduced in a split mode at a 1:3 ratio between the flame ionization detection (FID) and EAD. The column effluents were carried to male antennae with continuous, charcoal-filtered air flow at the rate of 400 mL/min via a CS-55 stimulus controller (Syntech, Buchenbach, Germany). FID and EAD signals were simultaneously recorded using GC–EAD Pro software (Syntech, Buchenbach, Germany).

### 2.4. Gas Chromatography–Mass Spectrometry (GC–MS)

Crude *A. strojny* pheromone gland extract was analyzed using GC–MS on an Agilent 7890/5977B instrument (Agilent Technologies, Santa Clara, CA, USA) equipped with an HP-5 column (30 m length × 250 µm inner diameter × 0.25 µm film thickness; J&W Scientific, Agilent Technologies, Santa Clara, CA, USA). The column temperature settings were the same as those used for the GC–EAD analysis. One microliter (equal to extracts from eight females) of crude pheromone gland extract was injected for analysis. Helium was used as the carrier gas at a constant flow rate of 1.2 mL/min, and the inlet was set at 280 °C with a splitless mode. The MS ionization voltage was 70 eV and the ion source temperature was 230 °C. The bioactive compounds were verified by comparison of the retention time and mass spectra with authentic standards.

For analysis of the dimethyl disulfide (DMDS) adducts of bioactive components, a high-polarity DB-23 column (30 m length × 250 µm inner diameter × 0.25 µm film thickness; J&W Scientific, Agilent Technologies, Santa Clara, CA, USA) was coupled to a GC–MS on an Agilent 7890/5977B instrument (Agilent Technologies, Santa Clara, CA, USA). The column oven temperature was maintained at 50 °C for 2 min and was then raised by 10 °C /min to 160 °C, and then 4 °C/min to 220 °C. The flow rate of the helium carrier gas was 1.0 mL/min, and the inlet was set at 230 °C with a splitless mode. The MS ionization voltage was set at 70 eV and the ion source temperature was 230 °C.

### 2.5. DMDS Derivatization of Pheromone Components

To determine the double bond positions in candidate monoenyl sex pheromone compounds, the pheromone extract was treated with DMDS prior to GC–MS analysis. DMDS derivatization was conducted as described by Vang et al. [26]. First, hexane was removed from the crude female pheromone gland extract under a constant nitrogen stream. Then the residues were treated with 50 µL of DMDS supplemented with 5 µL of a diethyl ether solution of iodine (60 mg/mL) and held at 40 °C overnight [27]. Afterward, 100 µL of 5% sodium thiosulfate solution was added to the aforementioned solution. Finally, the DMDS derivatives were extracted with 200 µL of n-hexane for GC–MS analysis.

### 2.6. Field Trapping

The field trapping experiment was carried out at the tea plantations in Longwu Tea Town, Hangzhou City, Zhejiang Province, China (30.17° N, 120.03° E) from 15 March to 2 April 2021. Synthetic pheromone compounds of *A. strojny* Z11-14:Ac and Z11-14:OH were prepared at a concentration of 10 µg/µL using n-hexane as a solvent. To prepare the lure, a total of 100 µL of the above two solutions was impregnated into white rubber septa at different ratios. Specifically, six different Z11-14:OH to Z11-14:Ac ratios, namely, 0:100, 5:95, 10:90, 20:80, 40:60, and 100:0, were prepared (Table 1), and each ratio had four replicates. A white rubber septum loaded with 100 µL of n-hexane was used as a blank control. The wing traps (27 cm length × 21 cm width × 14 cm height; Enjoy Technology Co., Ltd., Zhangzhou, China) baited with white rubber septa containing the corresponding blends of *A. strojny* pheromone compounds were used for field trapping. The traps were hung by a plastic pole at a height of 25 cm above the tea crop and randomly set in the tea plantation at 20-m intervals. All the trap catches were counted every three days, and the sticky board of each trap was changed after each count.

### 2.7. Chemicals

The Z11-14:Ac and Z11-14:OH (97%) standards were purchased from ZeQuan Bio-technology Co., Ltd. (Hangzhou, China), and 99.5% n-hexane was purchased from Tokyo Chemical Industry Co., Ltd. (Tokyo, Japan). Dimethyl disulfide (99.7%) was purchased from Sigma Aldrich Co., Ltd. (St. Louis, MO, USA). Sodium thiosulfate (99%) was provided by Aladdin Co., Ltd. (Shanghai, China).

### 2.8. Statistical Analysis

To ensure normal distribution and homogeneity of the data, the number of male moths caught in the field trapping assay was first subjected to log_10_ (catch number + 0.5) transformation, and then analyzed using one-way ANOVA followed by Tukey’s post hoc test (*p* < 0.05). All the data were analyzed using IBM SPSS Statistics 21.0 (IBM Corporation, Armonk, NY, USA).

## 3. Results

### 3.1. Male A. strojny Antennae Responded to Two Bioactive Components in Female Gland Extract

**Figure 2:**
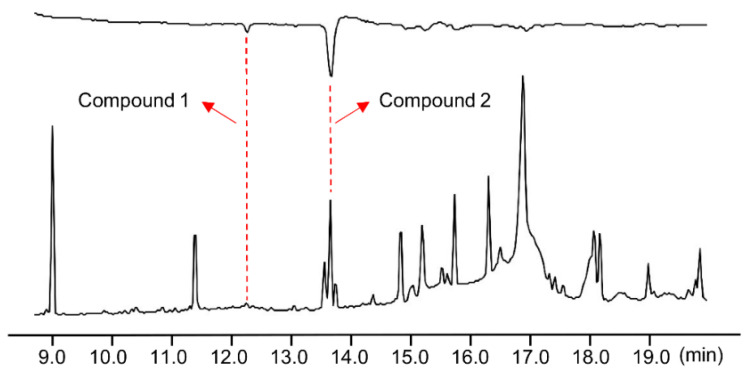
Gas chromatography–electroantennographic detector (GC–EAD) recording of *Archips strojny* male moth antennal responses to female crude gland extract (upper: EAD, lower: GC). Two components (Compounds 1 and 2) from the *A. strojny* female pheromone gland extract with retention times of 12.25 min and 13.67 min, respectively, were detected from active responses by the male moth antennae.

GC–EAD analysis of a crude *A. strojny* female gland extract revealed two bioactive compounds that elicited a male antennal response. As shown in Figure 2, the retention times (RTs) of these two bioactive compounds were 12.25 min (Compound 1) and 13.67 min (Compound 2). In terms of the relative amount, the GC chromatogram indicated that Compound 2 has a much higher relative abundance compared with Compound 1 in the crude pheromone gland extract. Therefore, Compound 2 was assumed to serve as the main sex pheromone component in *A. strojny*, while Compound 1 was a minor component.

### 3.2. Chemical Structures of Bioactive Compounds 1 and 2

**Figure 3:**
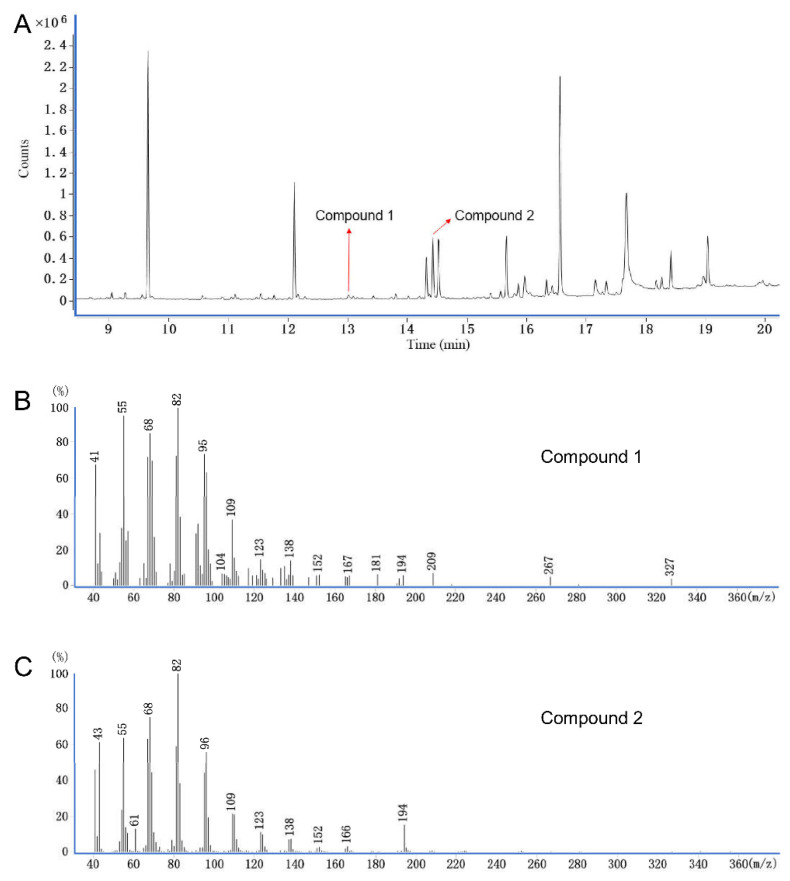
Gas chromatography–mass spectrometry (GC–MS) analysis of pooled *Archips strojny* female moth crude pheromone gland extract (n = 8). (**A**) Total ion chromatogram (TIC), (**B**) mass spectrum of Compound 1 ((Z)-11-tetradecenyl alcohol (Z11-14:OH), retention time (RT) 13.01 min), (**C**) mass spectrum of Compound 2 ((Z)-11-tetradecenyl acetate (Z11-14:Ac), RT 14.43 min).

The pheromone gland extract of *A. strojny* females was further analyzed to determine the chemical structures of the bioactive compounds (Figure 3). As indicated, the two EAD-active components were recorded on a total ion chromatogram (TIC) with RTs of 13.01 min (Compound 1) and 14.43 min (Compound 2). Specifically, the relative abundance of the two compounds in the pheromone was approximately 8:92 according to the calculated peak area (Figure 3A). As shown in Figure 3B,C, although the molecular ions (M^+^) were not detected in the mass spectra, the characteristic ions at *m/z* 194 indicated that these two compounds were either alcohol ([M − 18]^+^) or acetate ([M − 60]^+^) with a C14 chain [17]. The mass spectra (Figure 3C) showed a diagnostic ion at *m*/*z* 61, which is usually indicative of the presence of [AcOH + 1]^+^ [17]. Therefore, Compound 1 was estimated to be an unbranched tetradecenyl alcohol, and Compound 2 was the corresponding tetradecenyl acetate [17,26].

**Figure 4:**
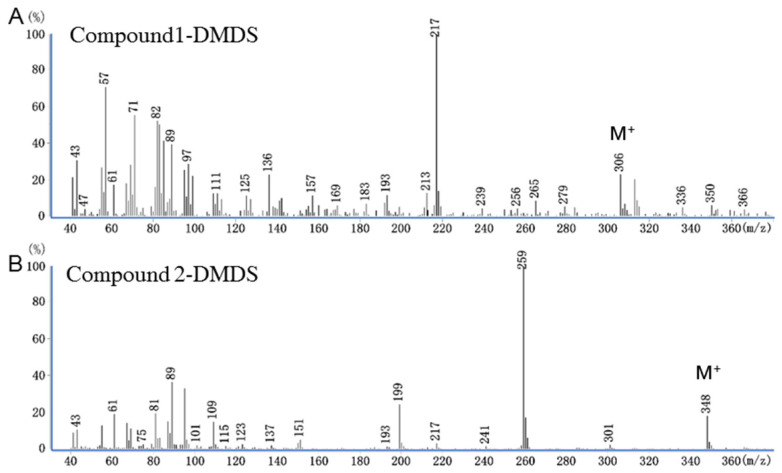
Gas chromatography–mass spectrometry (GC–MS) analysis of pooled *Archips strojny* female crude pheromone gland extract (n = 8) treated with dimethyl disulfide (DMDS). (**A**) Mass spectrum of Compound 1-DMDS adduct, (**B**) mass spectrum of Compound 2-DMDS adduct.

The GC–MS analysis of female crude extract treated with DMDS revealed that the adduct of Compound 1 exhibited M^+^ at 306 and diagnostic fragment ions at *m*/*z* 217 and 89 (Figure 4A), and the DMDS adduct of Compound 2 showed M^+^ at 348 and diagnostic fragment ions at *m*/*z* 259 and 89 (Figure 4B). The spectral information of the DMDS adducts indicated that the two thiomethyl groups (SCH_3_) are assumed to add the double bond at the same carbon 11 positions of both tetradecenyl alcohol and acetate [19,28,29].

To further confirm the configurations of these two candidate pheromone compounds, two columns with different polar properties were used. The RTs and Kovats index (KI) of natural sex pheromones and synthetic standards were compared. In natural extracts, Compound 1 was shown to have an RT of 18.29 min and a KI of 2214, and Compound 2 was shown to have an RT of 18.01 min and a KI of 2194 in the DB-23 column, whereas in the HP-5 column, the RT and KI were recorded as 12.98 min and 1678, respectively, for Compound 1, and 14.40 min and 1810, respectively, for Compound 2 (Table 2), which were the same as those of the standard compounds, except that a minor RT shift (0.01 min) was noticed for Compound 1. Minor shifts of RT and KI between standard and natural extracts are a common phenomenon that has been reported for various identified sex pheromone compounds [30]. The detailed RT and KI information of these compounds in both high-polarity DB-23 columns and low-polarity HP-5 columns (Table 2) validated Compound 1 as Z11-14:OH and Compound 2 as Z11-14:Ac.

### 3.3. Attraction of Males to Synthetic Lures in the Field

**Figure 5:**
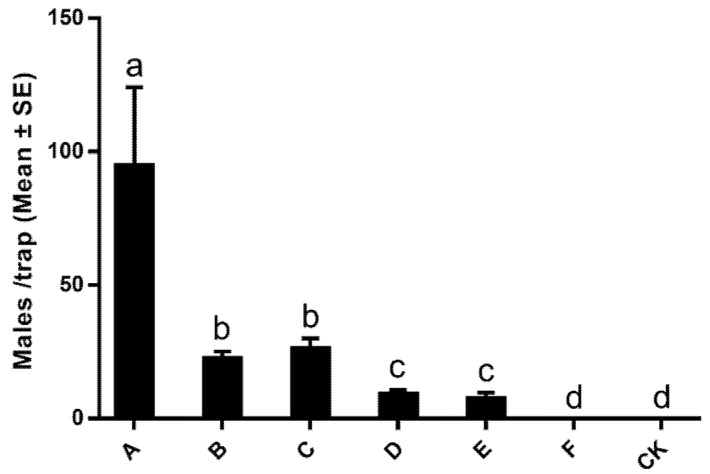
Numbers of *Archips strojny* male moths caught in traps baited with different ratios of synthetic sex pheromone components during a field trapping assay conducted at Longwu in Hangzhou City, Zhejiang Province, China (15 March 15–2 April 2021). A–F indicate different ratios of (Z)-11-tetradecenyl alcohol (Z11-14:OH) to (Z)-11-tetradecenyl acetate (Z11-14:Ac): (A) 0:100, (B) 5:95, (C) 10:90, (D) 20:80, (E) 40:60, and (F) 100: 0. CK indicates traps baited with only 100 µL of n-hexane. Four replicates were used for each ratio. Different letters above the bars indicate significant differences (*p* < 0.05, one-way ANOVA analysis followed by a Tukey’s post hoc test (*F* = 90.235; *df* = 6.21)).

Figure 5 shows the attractive effects of lures impregnated with different ratios of synthetic sex pheromone components in the Longwu tea plantations. Traps baited only with Z11-14:Ac captured the maximum number of males (94.50 ± 29.72 males per trap, mean ± SE, lure A in Figure 5), and traps impregnated with hexane solvent alone failed to attract any males (lure CK in Figure 5). Although the relative ratio of Z11-14:OH to Z11-14:Ac in lures B and C was designated based on the naturally occurring ratio of 8:92, when compared with lure A, both lures trapped significantly fewer male moths (lures B and C in Figure 5). With the increase in Z11-14:OH, the lures’ attractiveness to male moths was further diminished (lures D, E, and F in Figure 5). These results suggested that the dominant compound Z11-14:Ac acts as the attractant in the sex pheromone, while the minor compound Z11-14:OH seems to be a pheromone antagonist.

## 4. Discussion

To develop sex pheromone-based management strategies for *A. strojny* in Chinese tea plantations, we first analyzed the crude extracts from female *A. strojny* abdominal tips using GC–EAD; two EAD-active components (Compounds 1 and 2) were detected at a ratio of 8:92 (Figure 3). Compounds 1 and 2 were identified as 11-tetradecenyl alcohol and 11-tetradecenyl acetate via GC–MS analysis of the crude extracts and their corresponding DMDS derivatives. Furthermore, (*Z*)-configuration of the carbon 11-position double bonds was assigned for both sex pheromone components. Finally, the attractiveness to male *A. strojny* of these two components at different ratios was tested in the field trapping assays. The results suggested that the main component Z11-14:Ac played an important attractive role, while the minor component Z11-14:OH seemed to serve as a sex pheromone antagonist.

In addition to *A. strojny*, the sex pheromone components of eight other species of the genus *Archips* have also been elucidated, and Z11-14:Ac was identified as a common sex pheromone component for all of them [31]. The sharing of Z11-14:Ac as a sex pheromone component across different species in this genus indicates that Z11-14:Ac is an important compound for mating communication for *Archips* moths, and this knowledge will contribute to the development of Z11-14:Ac-based mating disruption techniques targeting all nine species. Like *A. strojny*, three of the eight species in this genus that have known sex pheromone compositions also use Z11-14:Ac as the predominant sex pheromone compound [31]. These three species are the variegated golden tortrix *Archips xylosteana* L., citrus leafroller *Archips atrolucens* Diakonoff, and the European leafroller *Archips rosana* L. [26,32,33]. Moreover, *A. rosana* and the fruit-tree tortrix *Archips podana* Scopoli are the only two reported species from this genus that also produce Z11-14:OH as a minor sex pheromone component, suggesting that *A. strojny* might have a close evolutionary relationship to *A. rosana* and *A. podana* in terms of sex pheromone production [33,34]. In addition to Z11-14:Ac and Z11-14:OH, *Archips* species also produce some other typical Type-I sex pheromone components, such as E11-14:Ac, Z9-14:Ac, 12:Ac, and 14:Ac [31]. The variation in sex pheromone compositions or ratios of the same sex pheromone components in *Archips* moths contributes indispensably to the establishment of the species-specific mating communication system.

The minor *A. strojny* sex pheromone compound Z11-14:OH was shown to play an antagonistic role in attracting male moths in the field trapping assay (Figure 5). This phenomenon seems unique; however, it is not an exception. In fact, research in the 1970s showed that moth species produced behaviorally inhibitory compounds [35,36]. Specifically, the female gypsy moth *Lymantria dispar* L. produces an olefin, 2-methyl-cis-7-octadecene, to mask the attractiveness of the sex pheromone to males [36], and the Eastern spruce budworm, *Choristoneura fumiferana* Clemens, releases E11-14:OH, the (*E*) configuration isomer of Z11-14:OH, to inhibit the attractive effect of its sex pheromone [35]. The most intensively investigated example is the inhibitory effect of Z11-16:OH in the cotton bollworm *Helicoverpa armigera* Hübner; Z11-16:OH has been found to reduce male attraction to pheromone lures in wind-tunnel assays at 5% and 15%, and in field bioassays at 6% of the major pheromone component [36,37,38,39,40]. It was hypothesized that the observed attractiveness-masking phenomenon was a result of immature female *H. armigera* modulating the attraction of males by emitting the antagonist Z11-16:OH along with the pheromone during the first two nights of calling and that this prevented males from mating with immature females, ensuring maximum fecundity [41]. In our study, we speculate that Z11-14:OH has a similar function as the inhibitor Z11-16:OH for *H. armigera*. Different pheromone odorant receptors are responsible for sensing Z11-14:OH and Z11-14:Ac in the sensilla trichodea of male antennae, and when the special odorant receptor senses Z11-14:OH, it inhibits other odorant receptor functions. Field-collected *A. strojny* samples with unknown specific ages were used for the pheromone extract; whether this species uses Z11-14:OH to signal their sexual maturity needs to be further investigated. Interestingly, for the tobacco budworm moth *Chloridea virescens* Fabricius, the addition of Z11–16:OH at a ratio of 1% of the major component resulted in a two-fold increase in male moths attracted to the blend; however, a ≥5% ratio of Z11-16:OH in the blend decreased the number of field-trapped male moths substantially [42]. This indicated that the ratio of minor to major components is crucial to trap efficiency. As only ≥5% of Z11-14:OH was tested in our field trap assay, the effects of lower dosages of Z11-14:OH on male *A. strojny* need to be further investigated.

Regardless of the role of Z11-14:OH in the breeding behavior of *A. strojny*, our report provides scientific support for developing effective sex pheromone-based control strategies for this species. The traps baited with only 1 mg of Z11-14:Ac exerted a promising level of attractiveness to male *A. strojny* and seem to be applicable as a monitoring or mass trapping tool for tea tortrix pest management programs. The finding that Z11-14:Ac is a shared sex pheromone component across different species in the genus *Archips* indicated that Z11-14:Ac could be used for developing mating disruption techniques targeting multiple leafroller species.

## 5. Conclusions

In this study, we identified two bioactive compounds, Z11-14:OH and Z11-14:Ac, in the sex pheromone of *A. strojny*. Z11-14:OH and Z11-14:Ac were detected at a ratio of 8:92, indicating that Z11-14:OH is a minor sex pheromone component and Z11-14:Ac is the dominant component. The attractiveness of the lures diminished sharply as the Z11-14:OH ratio in the binary blends increased in the field trapping assays, suggesting that Z11-14:Ac is the attractant in the sex pheromone, while Z11-14:OH seems to be a sex pheromone antagonist. Our study will promote the development of sex pheromones as integrated pest management tools for tea tortrix, and likely for other leafroller species.

Based on the results of this study, Z11-14:OH is expected to be developed as an insect behavioral regulator for male *A. strojny* moths to inhibit or disrupt the perception of female courtship signals. The strong attractant efficiency of Z11-14:Ac indicates that it can be applied as a pheromone lure for population monitoring or mass trapping. Furthermore, the most promising application is to develop an efficient and sustained release agent, which not only provides a sustainable control strategy for *A. strojny* but also disrupts the mating communication of the other eight related leafroller species that produce Z11-14:Ac as the main component of their sex pheromones.

## Figures and Tables

**Table 1:** *Archips strojny* sex pheromone lure baited with (Z)-11-tetradecenyl alcohol (Z11-14:OH) and (Z)-11-tetradecenyl acetate (Z11-14:Ac) for field trapping.

Treatment	Lure Composition (µg)	
	Z11-14:OH	Z11-14:Ac
A	0	1000
B	50	950
C	100	900
D	200	800
E	400	600
F	1000	0
CK	0	0

**Table 2:** Gas chromatography–mass spectrometry (GC–MS) analysis of natural pheromone extract of *Archips strojny* and synthetic (*E*) and (Z) configuration standards on different GC columns (RT: retention time; KI: Kovats index).

Compound	DB-23 GC Column		HP-5 GC Column	
	RT (min)	KI	RT (min)	KI
Compound 1	18.29	2214	12.98	1678
Compound 2	18.01	2194	14.40	1810
Z11-14:OH	18.28	2213	12.97	1678
E11-14:OH	17.93	2187	12.92	1673
Z11-14:Ac	18.01	2193	14.40	1810
E11-14:Ac	17.68	2168	14.35	1805

## Data Availability

Data available on request due to privacy restrictions. The data presented in this study are available on request from the corresponding author.
